# Reduced Frontal Brain Volume in Non-Treatment-Seeking Cocaine-Dependent Individuals: Exploring the Role of Impulsivity, Depression, and Smoking

**DOI:** 10.3389/fnhum.2014.00007

**Published:** 2014-01-17

**Authors:** Cleo L. Crunelle, Anne Marije Kaag, Guido van Wingen, Hanna E. van den Munkhof, Judith R. Homberg, Liesbeth Reneman, Wim van den Brink

**Affiliations:** ^1^Department of Psychiatry, Academic Medical Center, University of Amsterdam, Amsterdam, Netherlands; ^2^Toxicological Center, University of Antwerp, Antwerp, Belgium; ^3^Collaborative Antwerp Psychiatric Research Institute (CAPRI), University of Antwerp, Antwerp, Belgium; ^4^Department of Radiology, Academic Medical Center, University of Amsterdam, Amsterdam, Netherlands; ^5^Department of Cognitive Neuroscience, Centre for Neuroscience, Donders Institute for Brain, Cognition, and Behaviour, Radboud University Nijmegen Medical Center, Nijmegen, Netherlands

**Keywords:** cocaine dependence, drug abuse, voxel-based morphometry, frontal, depression, nicotine

## Abstract

In cocaine-dependent patients, gray matter (GM) volume reductions have been observed in the frontal lobes that are associated with the duration of cocaine use. Studies are mostly restricted to treatment-seekers and studies in non-treatment-seeking cocaine abusers are sparse. Here, we assessed GM volume differences between 30 non-treatment-seeking cocaine-dependent individuals and 33 non-drug using controls using voxel-based morphometry. Additionally, within the group of non-treatment-seeking cocaine-dependent individuals, we explored the role of frequently co-occurring features such as trait impulsivity (Barratt Impulsivity Scale, BIS), smoking, and depressive symptoms (Beck Depression Inventory), as well as the role of cocaine use duration, on frontal GM volume. Smaller GM volumes in non-treatment-seeking cocaine-dependent individuals were observed in the left middle frontal gyrus. Moreover, within the group of cocaine users, trait impulsivity was associated with reduced GM volume in the right orbitofrontal cortex, the left precentral gyrus, and the right superior frontal gyrus, whereas no effect of smoking severity, depressive symptoms, or duration of cocaine use was observed on regional GM volumes. Our data show an important association between trait impulsivity and frontal GM volumes in cocaine-dependent individuals. In contrast to previous studies with treatment-seeking cocaine-dependent patients, no significant effects of smoking severity, depressive symptoms, or duration of cocaine use on frontal GM volume were observed. Reduced frontal GM volumes in non-treatment-seeking cocaine-dependent subjects are associated with trait impulsivity and are not associated with co-occurring nicotine dependence or depression.

## Introduction

Impulsivity is an inherent feature of drug dependence and is closely related to frontal lobe function (Cho et al., 2013). In drug dependence, loss of control over drug use (driving the individual toward excessive drug use) and impulsiveness (leading to early relapse following periods of abstinence) are examples of related frontal lobe dysfunction. Smaller gray matter (GM) volumes in frontal cortical areas (in the middle frontal gyri and the orbito- and dorso-lateral frontal cortices) have been repeatedly reported in cocaine-dependent individuals compared to non-drug users (Liu et al., 1998; O’Neill et al., 2001; Fein et al., 2002; Franklin et al., 2002; Matochik et al., 2003; Sim et al., 2007; Lim et al., 2008; Tanabe et al., 2009; Alia-Klein et al., 2011; Weller et al., 2011; Ide et al., 2013). Reduction in the volume of frontal lobes [orbitofrontal cortex (OFC) and middle frontal gyrus] has also been associated with the duration of cocaine use (Barrós-Loscertales et al., 2011; Ersche et al., 2011) and with the measures of impulsive action and impulsive choice (Matsuo et al., 2009; Ersche et al., 2011; Moreno-López et al., 2012), inherent features associated with the frontal lobe dysfunction and increased in cocaine- and drug-dependent individuals (Prisciandaro et al., 2012). In the (medial) OFC, GM volume reductions were also observed in cocaine-dependent patients abstinent from 20 days (Matochik et al., 2003) up to 5 years (Tanabe et al., 2009), suggesting long-term brain volume abnormalities as a consequence of chronic cocaine use or as a characteristic inherently present in individuals vulnerable for cocaine dependence. The above mentioned studies were mainly performed in treatment-seeking cocaine-dependent patients (Fein et al., 2002; Franklin et al., 2002; Matochik et al., 2003; Tanabe et al., 2009; Barrós-Loscertales et al., 2011; Weller et al., 2011; Moreno-López et al., 2012; Ide et al., 2013) or in current cocaine abusers recruited via local advertisement or via unknown inclusion routes and thus without information regarding treatment-seeking status (Liu et al., 1998; Sim et al., 2007; Alia-Klein et al., 2011). Currently, only one study provides structural brain imaging data from identified non-treatment-seeking cocaine-dependent individuals (Ersche et al., 2011). However, we believe it is important to take the treatment-seeking condition into account, because treatment seeking is associated with severity and duration of dependence (Chitwood and Morningstar, 1985; Varney et al., 1995; Robson and Bruce, 1997), and the presence of more (severe) comorbid disorders (Regier et al., 1990) and more serious cognitive impairments and related social dysfunctions, which in themselves are also related to reductions in brain volume (Pennanen et al., 2005; Schiffer et al., 2010; van Wingen et al., 2013). Thus, investigating a population of non-treatment-seeking cocaine-dependent individuals provides data that are less influenced by the duration and the severity of addiction and by complex comorbidities than in treatment-seeking cocaine-dependent individuals.

Cocaine-dependent individuals are characterized by high levels of impulsivity, depressive symptoms, and comorbid drug use, especially smoking (Prisciandaro et al., 2012; Vonmoos et al., 2013). However, the combined influence of these variables on brain morphology in non-treatment-seekers has not been previously explored. A few studies have investigated the effects of impulsive traits (Ersche et al., 2011; Moreno-López et al., 2012), impulsive action and motor performance (Sim et al., 2007; Ersche et al., 2011) and decision making (Tanabe et al., 2009) on GM volume reductions in cocaine-dependent individuals and two studies assessed the additional effect of comorbid nicotine use on cocaine-related GM volume reductions (Sim et al., 2007; Alia-Klein et al., 2011), whereas no study simultaneously investigated the effects of depressive symptoms in these samples. These variables (i.e., impulsivity, smoking, and depressive symptoms) also correlate individually with volume reductions in frontal brain structures with correlations being most prominent for depression (Bora et al., 2012) and impulsivity (Cho et al., 2013). The correlation is less clear for smoking. Studies report reduced frontal volumes associated with nicotine dependence (Alia-Klein et al., 2011; Zhang et al., 2011), but these findings only held for the anterior cingulate cortex (ACC) in a meta-analysis covering nicotine structural imaging studies (Pan et al., 2013).

In this study, we investigate GM volume differences between non-treatment-seeking cocaine-dependent individuals and non-drug using controls. Additionally, in non-treatment-seeking cocaine-dependent individuals, we investigate the relationship between frontal GM volume and self-reported impulsivity, comorbid nicotine use and the presence of depressive symptoms, and we investigate the relationship between duration of cocaine use and frontal GM volume.

## Materials and Methods

### Subjects

Cocaine using and non-drug using control males (aged between 20 and 55 years old) were recruited between September 2011 and December 2012 through a local advertisement in the metropolitan area of Amsterdam (the Netherlands). Cocaine users only passed the screening if using cocaine regularly, i.e., using at least 1 g of cocaine per week during at least 12 consecutive months prior to inclusion. Control males were included when they have never used cocaine. Both the cocaine using and non-drug using males were asked to participate in this study during the same time period, and included (and scanned) within the same period. Participants from the cocaine using group were only invited to participate in the research and were not asked to initiate treatment. Alcohol and nicotine use were allowed in both groups. Subjects were excluded from study participation when currently taking medication, when having a prior or current diagnosed psychiatric (including drug dependence, except for nicotine dependence that was not an exclusion criterion) or neurological disorder, when having suffered head trauma, or when having metal in their body. Cocaine users were excluded if they reported having searched for treatment related to cocaine use prior to study inclusion. Cocaine was the primary preferred substance. All subjects were paid for their participation.

The study was approved by the Ethical Review Board of the Academic Medical Center of the University of Amsterdam, the Netherlands. All subjects gave written informed consent.

### Clinical assessments

Drug use (cocaine, alcohol, nicotine, cannabis, ecstasy, speed, opiates, and sedatives) was documented prior to study inclusion using in-house drug use questionnaires and cocaine dependence was diagnosed upon study inclusion according to DSM-IV criteria using the Mini International Neuropsychiatric Interview (MINI; Sheehan et al., 1998). Only regular cocaine users including the criteria for current DSM-IV cocaine dependence and not meeting criteria for any other substance dependence diagnosis were included in the study.

Trait impulsivity was assessed using the Barratt Impulsivity Scale (BIS; Patton et al., 1995) and the three main dimensions including motor, non-planning, and attentional impulsivity were computed. Nicotine dependency scores were assessed using the Fagerström Test for Nicotine Dependence (FTND; Heatherton et al., 1991). Depressive symptoms were assessed using the Beck Depression Inventory (BDI; Beck and Steer, 1987). Subjects were additionally characterized on the levels of premorbid intelligence [IQ; using the Dutch variant of the National Adult Reading Test (DART); Schmand et al., 1991].

### Image acquisition

Images were acquired on a 3.0-T whole body MR scanner (Philips Achieva) with a 32 channel SENSE head coil. Three-dimensional T1-weighted images with the following parameters: repetition time (TR) = 8.24 ms, echo time (TE) = 3.79 ms, flip angle = 8°, slice thickness = 1 mm, scan resolution = 240 mm × 240 mm, field-of-view (anterior–posterior/ feet–head/right–left) = 240/240/220 mm, and voxel size = 1 mm^3^ were obtained for voxel-based morphometry (VBM) analysis. The head was stabilized with foam for minimal head movement.

### Image processing

For detection of structural brain differences, the VBM8 toolbox embedded in SPM8 (Statistical Parametric Mapping; Wellcome Department of Cognitive Neurology, London, UK) and running on Matlab 2012b (The MathWorks Inc., Natick, MA, USA) was used. Scans were manually checked for movement and ringing-like artifacts. Images were segmented into gray and white matter (WM), spatially normalized to MNI space using the high-dimensional DARTEL template (Ashburner, 2007) and resampled into 1.5 mm isotropic voxels corrected for global brain volume using non-linear modulation. Hidden Markov Random Fields (HMRF) of 0.15 and a multi-threaded Spatial Adaptive Non-Local Means (SANLM) de-noising filter were applied to remove spatial noise. Smoothing was performed with an 8-mm Full Width at Half Maximum (FWHM) Gaussian kernel and the smoothed GM images were used for statistical analysis.

### Statistical analysis

Group differences in questionnaire scores were assessed using independent-samples *T*-tests or non-parametric tests when appropriate in SPSS v20 (Statistical Package for the Social Sciences). Categorical data were analyzed using Chi-square tests in SPSS v20. Correlations between clinical assessments were analyzed using partial correlations with age as a covariate. Data are presented as means ± standard deviations (SD) or as median ± interquartile range where appropriate. A *p*-value <0.05 was considered statistically significant.

Gray matter images were analyzed using voxel-wise statistical tests in SPM8 with age as covariate. Differences between cocaine-dependent subjects (COC) and non-drug using healthy controls (HCs) were assessed using ANCOVA. Correlations within the COC group between brain volume and impulsivity subscores, nicotine and depression scores, and duration of cocaine use were assessed using multiple regressions. Four separate models were used to assess the effects of (1) the three impulsivity subscores, (2) FTND, (3) BDI, and (4) duration of cocaine use on regional frontal volume separately. Another model included all questionnaires (i.e., BIS, BDI, and FTND) to assess whether possible correlations remained significant after correction of all other measures. All voxel-wise statistical tests were family wise error (FWE) corrected (*p* < 0.05) for multiple comparisons across the whole-brain or the region-of-interest (ROI) using a small volume correction (SVC; Worsley et al., 1996) at the cluster level with an initial height threshold of *p* < 0.001 uncorrected and non-stationary correction (Hayasaka et al., 2004). Because of our *a priori* hypothesis about the frontal cortex, we defined a frontal ROI using the Automated Anatomic Labeling atlas (AAL; Tzourio-Mazoyer et al., 2002) of the WFU Pickatlas toolbox (Maldjian et al., 2003, 2004) containing all prefrontal brain regions, including the ACC.

To explore whether differences in GM were accompanied with differences in WM, we also performed the same analyses with the WM images.

## Results

### Subjects and clinical assessments

A total of 30 COC and 33 HC were included and all participated in the study. Subjects were of similar age (COC: 32 ± 9 years; HC: 33 ± 9 years; *p* = 0.733) and IQ (COC: 103 ± 8; HC: 106 ± 9; *p* = 0.196). Cocaine users reported higher BIS impulsivity scores in all three dimensions (see Table 1) and higher BDI depression scores (COC: 11 ± 6; HC: 3 ± 5; *p* < 0.001) compared to HCs. Smokers in both groups did not differ in mean FTND nicotine dependency scores (COC: 5 ± 2; HC: 4 ± 2; *p* = 0.444). However, there were significantly more smokers in the COC compared to the HC group (COC: 80% smokers; HC: 15% smokers; *p* < 0.001). Similar proportions of alcohol consumers were found in both groups (COC: 87% alcohol consumers; HC: 83% alcohol consumers; *p* = 0.430), but regular cocaine users drank more alcohol weekly compared to HCs (COC: 163 ± 131 g; HC: 82 ± 80 g; *p* = 0.009). There was no use of illegal substances in the HC group. In contrast, the COC group included recreational cannabis- (40%), ecstasy- (33%), amphetamine- (20%), and sedative-users (7%). Regular cocaine users used cocaine during an average of 7 ± 5 consecutive years and an average of 2 ± 1 days/week with a mean of 2.6 ± 1.9 g of cocaine per session.

**Table 1:** **Trait impulsivity scores as assessed using the Barratt Impulsivity Scale (BIS), including the three main dimensions: attentional impulsiveness, motor impulsiveness, and non-planning impulsiveness**.

BIS scores	COC (*n* = 30)	HC (*n* = 33)	*p*-Value
Total	73.8 ± 7.8	59.4 ± 6.9	<0.001
Attentional impulsiveness	17.7 ± 5.1	11.9 ± 3.6	<0.001
Motor impulsiveness	24.5 ± 6.5	20.5 ± 4.1	0.005
Non-planning impulsiveness	28.5 ± 4.4	15.5 ± 5.4	<0.001

*Data are presented as mean ± standard deviation. A *p*-value <0.05 was considered significant*.

### VBM group difference analysis

**Figure 1:**
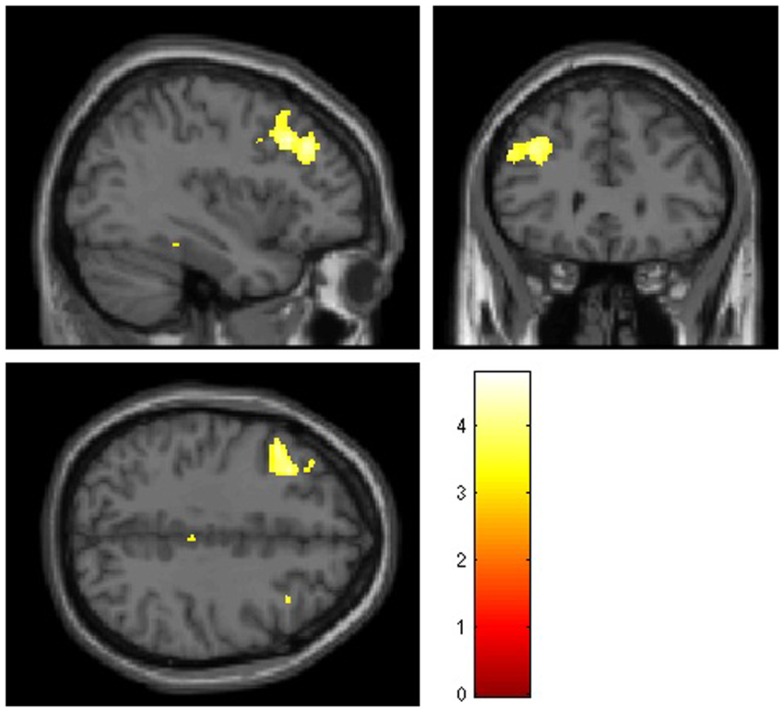
**Mean smaller gray matter volumes observed in 30 non-treatment-seeking cocaine-dependent individuals compared to 33 non-drug using controls in the left middle frontal gyrus using whole-brain voxel-based morphometry analysis**. Significant clusters (*p* < 0.05 corrected, at a height threshold of *p* < 0.001, uncorrected) are shown on an underlying Montreal Neurological Institute (MNI) template brain (left = left). Statistical tests between groups were performed using ANVOCA covariated for age in SPM8. The red/orange scale represents the corresponding *T* values.

Voxel-wise group comparisons revealed smaller GM volumes in cocaine users compared to controls only in the left middle frontal gyrus (coordinates *x y z* = −33 23 37; *k* = 1404; *Z* = 4.39; *p*_(FWE)_ = 0.002; see Figure 1). No larger regional GM volumes were observed in COC compared to HC. Also, there were no WM volume differences between non-treatment-seeking cocaine-dependent individuals and non-drug using controls.

### Correlation analyses in non-treatment-seeking cocaine-dependent individuals

In non-treatment-seeking cocaine-dependent individuals, no significant correlations were found between BIS subscale scores and duration of cocaine use (all *r* < 0.17; all *p* > 0.391), BDI (all *r* < 0.21; all *p* > 0.261), or FTND (all *r* < 0.31; all *p* > 0.147). Also, BIS subscale scores were not significantly inter-correlated (all *r* < 0.18; all *p* > 0.340).

Voxel-wise correlations with GM volume showed that attentional impulsiveness was significantly negatively associated with the right OFC (encompassing the superior, middle, and inferior gyri; coordinates *x y z* = 21 38 −14; *k* = 562; *Z* = 4.13; *p*_(FWE)_ = 0.028) and the left precentral gyrus (coordinates *x y z* = −41 0 48; *k* = 352; *Z* = 3.87; *p*_(FWE)_ = 0.029), whereas a positive correlation was observed between motor impulsiveness and volume of the right superior frontal gyrus coordinates (coordinates *x y z* = 18 41 48; *k* = 646; *Z* = 4.50; *p*_(SVC)_ = 0.038). Measures of non-planning impulsiveness were positively associated with volume in the right inferior parietal gyrus (coordinates *x y z* = 51 −51 52; *k* = 415; *Z* = 4.85; *p*_(FWE)_ = 0.040).

**Figure 2:**
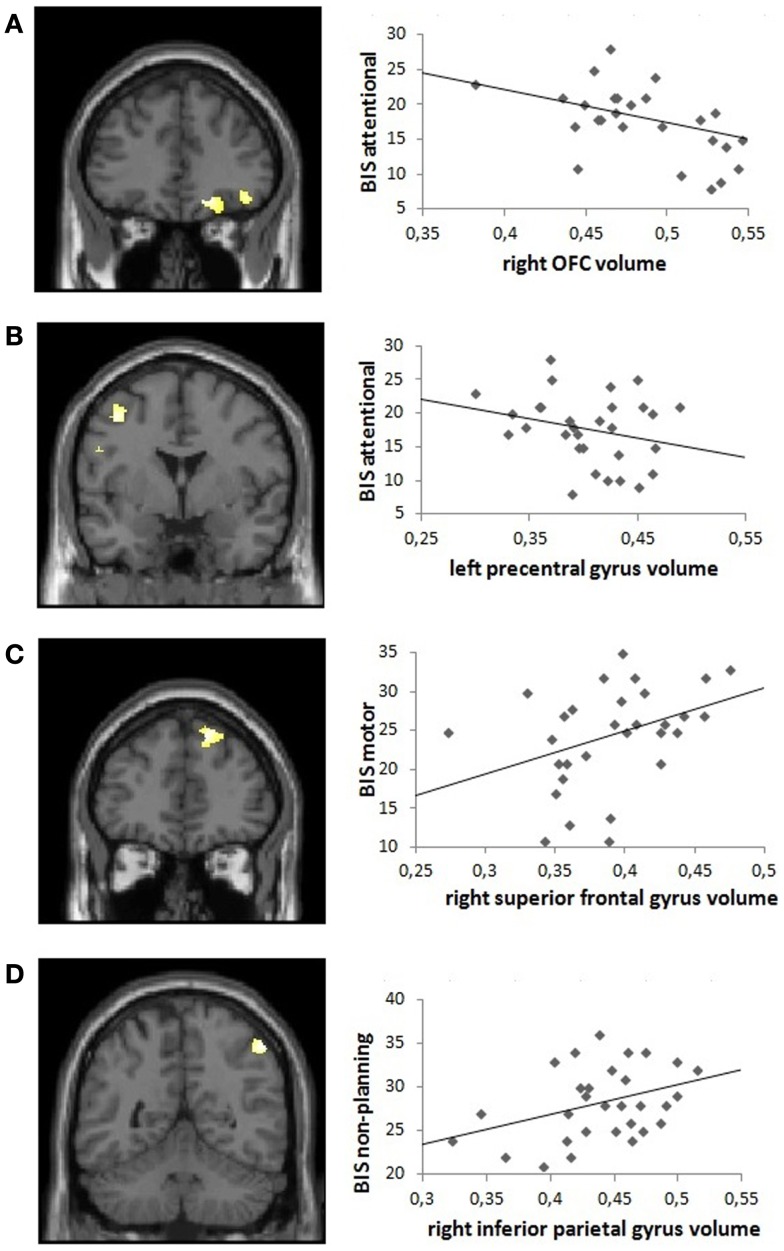
**Visual representation of the correlation between Barratt Impulsivity Scale (BIS) subscores and frontal gray matter volume in non-treatment-seeking cocaine-dependent individuals: positive correlations are shown between (A) BIS attentional impulsivity and right orbitofrontal cortex volume and (B) BIS attentional impulsivity and left precentral gyrus volume, and negative correlations between (C) BIS motor impulsivity and right superior frontal gyrus volume and (D) BIS non-planning impulsivity and right inferior parietal gyrus volume, derived from the model including both BDI and FTND covariates**. Left panels: coronal sections illustrating the significant clusters (*p* < 0.05 corrected, at a height threshold of *p* < 0.001, uncorrected). Right panels: scatter plots illustrating the correlations in the anatomically defined regions of interest.

Fagerström test for nicotine dependence scores (nicotine dependency) and BDI scores (depressive symptoms) were not correlated with GM volumes in non-treatment-seeking cocaine-dependent individuals, and BIS attentional impulsiveness and GM volume remained significantly correlated after controlling for BDI and FTND scores in the right OFC (inferior and superior gyri; coordinates *x y z* = −27 26 −15; *k* = 158; *Z* = 3.73; *p*_(SVC)_ = 0.020) and in the left precentral gyrus (coordinates *x y z* = −39 0 48; *k* = 301; *Z* = 3.97; *p*_(FWE)_ = 0.040). The positive correlation between motor impulsiveness and volume in the right superior frontal gyrus also remained significant (coordinates *x y z* = 17 41 48; *k* = 646; *Z* = 4.25; *p*_(FWE)_ = 0.044). For non-planning impulsiveness, a trend remained with GM volume correlation in the right inferior parietal gyrus (coordinates *x y z* = 51 −52 54; *k* = 338; *Z* = 4.62; *p*_(FWE)_ = 0.082) (Figure 2).

No significant associations between duration of cocaine use and frontal GM volume were observed.

## Discussion

In non-treatment-seeking cocaine-dependent individuals compared to non-drug using HCs, significant GM volume reductions were found in the left middle frontal gyrus. Right OFC and left precentral gyrus volumes were negatively correlated with attentional impulsiveness. Motor impulsiveness was positively correlated with superior frontal gyrus volume and non-planning impulsiveness with volume in the right inferior parietal gyrus. No significant correlation was observed with nicotine dependence or depressive symptoms, suggesting that the association between frontal GM and trait impulsivity is little influenced by smoking severity and depressive symptoms. Finally, no significant associations between duration of cocaine use and frontal GM volume were observed.

The findings of smaller frontal GM volume (in the middle frontal gyrus) in non-treatment-seeking cocaine-dependent individuals compared to non-drug using controls is in correspondence with the current literature of reduced frontal GM volume in treatment-seeking cocaine-dependent patients (Matochik et al., 2003; Tanabe et al., 2009; Moreno-López et al., 2012). The most consistent findings of frontal GM differences in cocaine-dependent patients compared to controls are smaller volumes in (medial) OFC, which we did not replicate in this sample of non-treatment-seekers compared to non-drug using controls. However, smaller (medial, inferior, and superior) OFC brain volume significantly correlated with measures of (attentional) impulsivity. These findings are in line with the current literature observing correlations between OFC volume and impulsivity in treatment-seeking cocaine-dependent patients (Matsuo et al., 2009; Tanabe et al., 2009), in non-treatment-seeking cocaine users (Ersche et al., 2011), and in non-drug users (Cho et al., 2013).

It has been proposed that greater volumes of OFC and PFC imply greater trait impulsivity (measured using the BIS) in healthy subjects (Cho et al., 2013), but negative correlations between OFC volume and BIS trait impulsivity have been hypothesized and documented as well (Matsuo et al., 2009). A study assessing impulsivity in cocaine-dependent patients found a positive association between frontal volume and trait impulsiveness (measured using the UPPS) in cocaine-dependent patients, but an opposite pattern in non-drug using HCs (Moreno-López et al., 2012). In contrast, we observed a negative correlation between frontal GM volumes and attentional impulsiveness in non-treatment-seeking cocaine-dependent individuals and a positive correlation between frontal GM volume and motor- and non-planning impulsiveness. This could indicate that, in non-treatment-seeking cocaine-dependent patients, the associations between frontal GM volume and impulsiveness are more closely associated with brain morphometry of high-impulsive individuals compared to brain morphology of treatment-seeking cocaine-dependent patients. Also, as presented in this study, frontal GM volume correlated either negatively (attentional impulsivity) or positively (e.g., for motor impulsivity) with BIS subscale scores in non-treatment-seeking cocaine-dependent individuals. Different prefrontal cortical regions are thought to be preferentially involved in complementary aspects of cognitive control to produce appropriate behavior (Ridderinkhof et al., 2004). The negative association between attentional impulsiveness and OFC and precentral gyrus volume suggests that these brain regions may be particularly involved in the cognitive control of behavior in cocaine dependence, whereas the positive association between motor impulsiveness and superior frontal gyrus volume suggests that this brain region may be particularly involved in the behavioral aspect of impulsiveness. This proposes a neurobiological dissociation between measures of impulsiveness, as indicated by a lack of correlation between the three BIS subscale scores observed here and by separate studies that do not observe correlations between BIS scores and neuropsychological cognitive and motor impulsivity tasks (Broos et al., 2012; Crunelle et al., 2013). It should be mentioned that the correlation between right inferior parietal gyrus GM volume and non-planning impulsivity did not remain significant after controlling for smoking severity and depressive symptoms. However, a trend remained, suggesting an influence of smoking and/or depressive symptoms on GM volume in the parietal lobule, which should be investigated further.

No effect of smoking comorbidity or depressive symptoms on frontal GM volume was observed in this sample of non-treatment-seeking cocaine-dependent individuals, in contrast to previous reports that observed volume reductions in frontal lobes associated with smoking and depression. This study does not exclude the possibility that comorbid nicotine use and depressive symptoms account for further frontal GM volume reduction; however, these might be especially important with increased severity and duration of dependence symptoms such that additional reductions may only occur in subjects with an extended duration and/or severity of addiction symptoms.

We were not able to demonstrate an association between frontal GM volume and duration of cocaine use. One could hypothesize that frontal volume reduction is part of a primary feature of high-impulsive individuals and an inherent risk factor for the development of cocaine dependence rather than a direct consequence of cocaine use or cocaine dependence. However, one cannot exclude a further influence of cocaine use on a further reduction of frontal GM volume when duration and/or severity of dependence is extended, which might additionally contribute to the more severe symptoms of dependence, cognitive impairments, and comorbidities observed in, e.g., treatment-seeking cocaine-dependent individuals. While this remains a largely unresolved question, in studies in treatment-seeking cocaine abusers using cocaine for approximately 10 years or longer, duration of cocaine use was associated with ACC, OFC, insula, and striatal volume reductions (Ide et al., 2013). This correlation between GM volume and duration of cocaine use was also observed in a recent study in non-treatment-seeking cocaine-dependent subjects (Ersche et al., 2011). This correlation is likely lacking in our study due to our smaller sample size or due to lower exposure (smaller range in years that cocaine was used; 7 ± 5 vs. 10 ± 7) compared to Ersche et al. Some studies propose that reduction of WM, but not GM, correlates with duration of cocaine use (O’Neill et al., 2001; Matochik et al., 2003). However, we were unable to replicate these findings in non-treatment-seeking cocaine-dependent individuals.

This study includes a well-defined population of non-treatment-seeking cocaine-dependent individuals. As a result, it provides valuable information on a group of cocaine-dependent individuals who are often understudied. Limitations of this study should also be noted. Adding a group of treatment-seeking cocaine-dependent individuals could provide us with more information by a direct comparison of volume differences between treatment-seeking and non-treatment-seeking cocaine-dependent individuals and toward entangling the possible opposite correlation between impulsive traits and frontal GM volume, and this should be investigated in future studies. Also, individuals with major depressive disorder were excluded from participation, but, in future studies, it would be interesting to investigate the influence of the presence of MDD on brain volume in non-treatment-seeking cocaine-dependent individuals as well. Analysis of the association between ROI volume and impulsivity, smoking, and depression scores should then also be investigated in larger sample sizes in treatment-seekers compared to non-treatment-seekers, or in non-treatment-seekers compared to non-drug using controls. As such, replication of these findings is needed. Finally, in further studies toward GM volume reductions associated with nicotine dependence, the amount of cigarettes/nicotine used should be addressed instead of smoking severity alone.

In summary, this study provides evidence that frontal brain volume (middle frontal gyrus) is smaller in non-treatment-seeking cocaine-dependent individuals compared to non-drug using HCs, and that smaller volume in the OFC and precentral gyrus is associated with attentional impulsiveness. Hypothetically, structural differences may (at least partly) predate the development of cocaine dependence and be related to impulsive traits; however, this question remains largely unresolved. In conclusion, our data suggest that reduced frontal GM volumes in non-treatment-seeking cocaine-dependent subjects are associated with trait impulsivity and are not associated with co-occurring nicotine dependence or depression.

## Author Contributions

Data were obtained by Cleo L. Crunelle and Anne Marije Kaag, who prepared the first draft. Cleo L. Crunelle, Hanna E. van den Munkhof, and Guido van Wingen analyzed the data. Hanna E. van den Munkhof, Judith R. Homberg, Liesbeth Reneman, and Hanna E. van den Munkhof actively participated in writing and revising the manuscript for publication.

## Conflict of Interest Statement

The authors declare that the research was conducted in the absence of any commercial or financial relationships that could be construed as a potential conflict of interest. The editor Dr. Anna E. Goudriaan declares that, despite having collaborated with Dr. Van den Brink, the editing process was handled objectively and no conflict of interest exists.
